# Complete mitochondrial genome of the Mongolian lark, *Melanocorypha mongolica* (Aves: Passeriformes)

**DOI:** 10.1080/23802359.2017.1325340

**Published:** 2017-05-12

**Authors:** Zan Zeng, Jinyao Lu, Mengli Yang, Hui Kang, Bo Li

**Affiliations:** a College of Wildlife Resources, Northeast Forestry University, Harbin, China;; b State Forestry Administration Detecting Center of Wildlife, Harbin, China

**Keywords:** Sylvioidea, Alaudidae, Melanocorypha, mongolica, LA-PCR, mitochondrial, geneome

## Abstract

We sequenced the entire mitochondrial genome of *Melanocorypha mongolica* for the first time. The mitogenome is 17,358 bp in length, which includes 13 protein-coding genes, 22 tRNA genes, 2 ribosomal RNA (rRNA) genes, the control region (CR1), and the control region 2 (CR2). Gene order follows a pattern similar to those of Eurasian Skylark. Using mitochondrial genomes of *Melanocorypha mongolica* and other seven reference birds in Sylvioidea, we preformed Bayesian analysis based on concatenated protein-coding genes. The results reveal that Alaudidae and Acroccphalidae are clustered together, which is sister to the branches included Sylviidae and Leiothrichidae. Further sequencing of mitochondrial genomes in Alaudidae is useful to advance phylogenetic relationship of species in the family.

Mitochondrial genomes have become the most widely used molecular markers among a broad array of molecular evolutionary and phylogenetic studies (Rong et al. 2016). Several molecular phylogeny of the Alaudidae has been published, based on mitochondrial and nuclear sequences from mostly larks species (Tieleman et al. 2003; Alström et al. 2013). However, only one complete mitochondrial genome, Eurasian Skylark Alauda *arvensis* (under accession no.JQ322641, Qian et al. 2013), is available in GenBank. Further sequencing of mitochondrial genomes in the family Alaudidae is a priority in order to advance this field of research. In this paper, we sequenced the entire mitogenome of the Mongolian lark, *Melanocorypha mongolica* for the first time. As one of the family Alaudidae comprising 97 species in 21 genera (Spottiswoode et al. 2013), the bird is well known in China, Mongolia, and Russian Federation because of its beautiful song and local abundance in meadow and hills.

Using the Tissue Extraction Kit (AxyPrep DNA, Corning Inc. Wujiang, Jiangsu, China), total genomic DNA was extracted from about 100 mg of muscle conserved in The SFA Detecting Center of Wildlife, Harbin. The bird was collected from Inner Mongolia Manzhouli (about 49° 35'51" N, 117° 27'54" E). The complete mitochondrial genome was amplified by the long and accurate polymerase chain reaction (LA-PCR) with the nine pairs of primers. Purified and recovered PCR products were sequenced directly using the primers on an ABI 3730 DNA Analyzer (performed by the Haigene Biological company, Harbin, China). Consensus sequences were generated with SeqMan software (DNAStar Inc., Madison, WI). To characterize phylogenetic relationship in Sylvioidea, nucleotide sequences for 13 protein-coding genes from seven reference mt genomes were aligned using the ClustalW algorithm in MEGA6.06 (Tamura et al. 2011). Aligned sequences were concatenated, verified by eye, and subjected to phylogenetic analysis using MrBayes3.1.2 (Ronquist & Huelsenbeck 2003) with the best-fit nucleotide substitution model (GTR + I + G) selected by AIC in MrModeltest 2.3 (Nylander 2004).

We determined the mitochondrial genome of *Melanocorypha mongolica* to be 17,358 bp in length. The sequence is deposited in GenBank under accession no. KY887027. The mitochondrial genome includes 13 protein-coding genes, 22 tRNA genes, 2 ribosomal RNA (rRNA) genes, the control region (CR1), and the control region 2 (CR2). Gene order follows a similar pattern to those of Eurasian Skylark. The ATG start codon is used in all protein-coding genes except ND2 (GTG). The TAA stop codon is used in six protein-coding genes except ND1(AGA), ND5 (AGA), COX1(AGG), Cytb (TAG). Incomplete stop codons T–– or TA– appears in COX3, ND4, and ND2. Two control regions (CRs) are 1146 and 656 bp in length, respectively. The order of two CRs is tRNA-Thr/CR1/tRNA-Pro/ND6/tRNA-Glu/CR2/tRNA-Phe, which is identical with those of Eurasian Skylark. Nucleotide composition of the entire mitochondrial genome is A: 29.74%, T: 23.02%, C: 32.76%, and G: 14.48%, with a higher AT content.

Bayesian inference tree obtained from the analysis of concatenated protein-coding genes in Sylvioidea is shown in Figure 1 and Bayesian posterior probabilities (BPP) for all branches are high (88–100%). We can see from the figure that the two species of Alaudidae are mostly close to each other. In Sylvioidea, Alaudidae and Acroccphalidae are clustered together, which is sister to the branches included in Sylviidae and Leiothrichidae. This phylogenetic relationship is similar to the results of previous researches (Qian et al. 2013).

**Figure 1.**
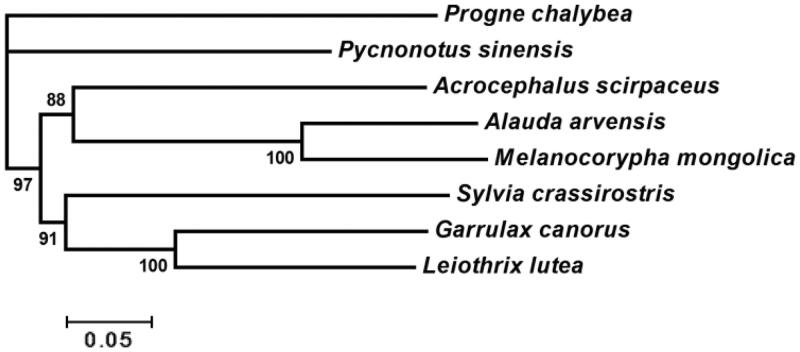
Bayesian inference tree from analysis of concatenated protein-coding genes in Sylvioidea. *Progne chalybea* is used as an outgroup. GenBank accession numbers for each species: *Progne chalybea* NC020605, *Pycnonotus sinensis* NC013838, *Acrocephalus scirpaceus* NC010227, *Alauda arvensis* NC020425, *Sylvia crassirostris* AM889141, *Garrulax canorus* KT633399 and *Leiothrix lutea* NC020427.

## References

[CIT0001] Alström P, Barnes KN, Olsson U, Barker FK, Bloomer P, Khan AA, Qureshi MA, Guillaumet A, Crochet P, Ryan PG. 2013 Multilocus phylogeny of the avian family Alaudidae (larks) reveals complex morphological evolution, non-monophyletic genera and hidden species diversity. Mol Phylogenet Evol. 69:1043–1056. 23792153 10.1016/j.ympev.2013.06.005

[CIT0002] Qian C. 2013 The sequencing and analysis of the complete mitochondrial genomes of 13 Passeriformes species. WuHu: Anhui Normal University Press.

[CIT0003] Qian C, Wang Y, Guo Z, Yang J, Kan X. 2013 Complete mitochondrial genome of Skylark, *Alauda arvensis* (Aves: Passeriformes): the first representative of the family Alaudidae with two extensive heteroplasmic control regions. Mitochondrial DNA. 24:246–248. 23311453 10.3109/19401736.2012.752481

[CIT0004] Nylander JAA. 2004 MrModeltest v 2. Program distributed by the author. Evolutionary Biology Centre, Uppsala University. Uppsala: Uppsala University Available from: http://scholar.google.com/scholar?hl=en&btnG=Search&q=intitle:MrModeltest#0

[CIT0005] Rong R, Li B, Li F. 2016 The complete mitochondrial genome of Japanese Marsh Warbler, *Locustella pryeri*. Mitochondrial DNA. Part A. 27:373–374. 10.3109/19401736.2014.895992 24617481

[CIT0006] Ronquist F, Huelsenbeck JP. 2003 MrBayes 3: Bayesian phylogenetic inference under mixed models. Bioinformatics. 19:1572–1574. 12912839 10.1093/bioinformatics/btg180

[CIT0007] Spottiswoode CN, Olsson U, Mills MSL, Cohen C, Francis JE, Toye N, Hoddinott D, Dagne A, Wood C, Donald PF, et al 2013 Rediscovery of a long-lost lark reveals the conspecificity of endangered Heteromirafra populations in the Horn of Africa. J Ornithol. 154:813–825.

[CIT0008] Tamura K, Peterson D, Peterson N, Stecher G, Nei M, Kumar S. 2011 MEGA5: molecular evolutionary genetics analysis using maximum likelihood, evolutionary distance, and maximum parsimony methods . Mol Biol Evol. 28:2731–2739. 21546353 10.1093/molbev/msr121 PMC3203626

[CIT0009] Tieleman BI, Williams JB, Bloomer P. 2003 Adaptation of metabolism and evaporative water loss along an aridity gradient. Proc Biol Sci. 270:207–214. 12590762 10.1098/rspb.2002.2205 PMC1691220

